# Improving the Ethical Permissibility of Medical Electives in Lower‐Resource Settings

**DOI:** 10.1111/dewb.70024

**Published:** 2026-01-19

**Authors:** Simon Paul Jenkins

**Affiliations:** ^1^ University of Warwick Medical School Coventry West Midlands UK of Great Britain and Northern Ireland

**Keywords:** Electives, ethics, global health, justice, low‐resource settings, medical education

## Abstract

This paper presents a moral‐theoretical evaluation of medical electives, applying different frameworks of distributive justice to the phenomenon of healthcare students visiting countries with less access to resources in order to bolster their own learning. Currently, discussions of ethical issues around these medical electives largely focuses on issues arising during the placement experience itself, in the form of dilemmas to which an individual must react in an ethical way. Applying different frameworks of distributive justice to the wider issue of how an elective project is designed and organised suggests that electives need to focus a great deal more on conferring benefits to host communities – that is, the communities being visited by students from wealthier settings – than is currently the case. To avoid the charge that medical electives are impermissibly extractive insofar as they impose burdens on host communities that are not justified by appeal to the benefits that the visiting students (or their communities) enjoy, I propose that both students and educators in the healthcare professions consider advocating an offsetting system to compensate host communities for the harms caused by the elective's taking place. The offsetting could comprise a monetary payment, a resolution to work in a lower‐resource setting in the future, or some combination of those two things. While this system may not even be permissible under all the distributive justice frameworks considered, it represents an improvement on the current state of affairs whilst still allowing trainees to gain useful experiences from electives.

## 1 Introduction

Students who are training for healthcare professions like medicine often undertake placements in different healthcare settings, commonly referred to as “electives”. Often, a student will do their elective in a lower‐resource setting than the one in which they are living and studying, for example travelling from Europe to sub‐Saharan Africa to study and gain experience in a new clinical environment there. This paper argues that in many cases, these experiences impose unjustified burdens on the communities that students visit on their electives, and proposes some structural and individualistic measures that could be taken to right these wrongs. I contend that the burdens placed on host institutions during these placements are under‐considered, but that they can be justified in either of two ways: A) monetary payment is made to offset the resource burden imposed by the medical student's visit, or B) the student uses their learning to provide some benefit, possibly to similar communities, in the future.

### 1.1 The Current State of Elective Ethics

Evidence from the literature suggests that medical electives have positive results for learners [1] – the students who go away on the placements. While ethical concerns surrounding electives are well‐documented, much of the ethics literature on these placements has so far focussed on ethical challenges that arise for students *during their elective* [2, 3, 4, 5, 6] like students being asked to perform procedures that might be deemed beyond their clinical competency at home. The general response to dilemmas of this type is that appropriate pre‐departure training, including learning that aims to develop cultural awareness, can mitigate the moral distress and discomfort that students can feel in these settings [7]. Including global health ethics content in this training can help students to respond appropriately to dilemmas that arise, helping them to consider problems in terms of tensions between different ethical commitments such as ensuring a good balance of harms and benefits, respecting patient autonomy, and other concepts like equality.

However, sometimes an elective is morally problematic *by design*: it involves ethical issues that are inherent in the elective project and its structure, and cannot be mitigated by developing students’ ethical responsiveness before departure [8]. These ethical issues are *necessary* parts of the elective; that is, the elective experience would not exist in its current form if these problems were removed. These issues have received less attention in the literature. They raise troubling questions that may not be resolvable by pre‐departure training, and may require a more wholesale overhaul of the concept of medical electives and how they are organised and conceived. Electives that are problematic in this way are effectively those that become ethically unsalvageable at an earlier stage: they sooner reach a point where no action can be taken that will rectify the problem, because the problem is not one arising *during* the elective; the problem is built into the structure of the elective, and so the ethical concerns are not easily mitigated by an individual taking one course of action or another. The distinction is not perfect: above I described students unexpectedly being asked to perform procedures outside their clinical competency, and one study found that an alarmingly high number of medical students were travelling to African nations for this very purpose [9]. Here, the issue of remaining within one's clinical competencies can clearly be one that either arises incidentally, *or* is built into the elective by design. Nevertheless, the distinction is useful for the discussion going forward.

## 2 Ethical Analysis

### 2.1 Costs

The most obvious way that an elective can be unethical by design is likely to be associated with the costs imposed on the host community. For a student to have a successful elective experience, they must be inducted into the new clinical setting, and supervised by someone from the host institution as they go about their learning. This usually means that a clinician from a low‐resource setting must sacrifice some of their time to ensure that the visiting student is properly inducted into the clinical setting, and is then exposed to a variety of learning experiences. Staff from host institutions recognise the costs associated with this sacrifice [10], noting that it leads to “inefficiency,” [11] ultimately impacting on their capacity to provide care to patients in their local community [12].

These costs are inherent to the elective experience, i.e. they must be imposed in order for the elective to meet its aims, and there is no way for the elective to function without some version of them being in play: students cannot undertake their electives without somebody from the local community devoting time to explain features of the setting to them and how they will fit into it during their time there. To the extent that these costs are a moral problem, then, the elective project is morally problematic by design. It may go without saying that the problem is likely to be worse the bigger the gulf between the settings in terms of their access to resources. A student travelling from the UK to Eastern Europe may face a problem of the same shape, but of a different size, from a student travelling from the UK to sub‐Saharan Africa.

These costs are at least a *prima facie* moral problem. They take resources from places that are – by definition – lower on resources to begin with. At their worst, then, electives in lower‐resource settings are an exploitative system of extracting resources from communities that are already under‐resourced, using up the valuable and scarce time of local clinicians in a way that harms their patients, for the educational benefit of someone from a well‐resourced community who is likely to use that benefit to serve their own community's needs. Such a damning potential criticism requires that we consider more seriously how this problem can be mitigated, and I follow Willott et al. in their view that current guidelines and recommendations for structuring and designing elective experiences [13, 14, 15, 16, 17] do not go far enough [18].

### 2.2 Justifying Costs

As a general principle, it is at least sometimes possible to justify the imposition of costs and harms. A surgeon imposes a harm by cutting into their patient, but this is justified by the overall benefit of the surgery. One question, then, is whether electives provide such a net benefit, so that the costs imposed on the host community are outweighed by the benefits accrued.

As I have said, the benefits of electives are manifold. Students report enjoying them, and can use them as an opportunity to complement their learning by developing an understanding of how healthcare works in a different setting, giving them a useful comparison piece for reflection, potentially providing opportunities to see diseases and conditions they do not see at home. Those from host institutions also report positives, noting that they learn from the students, and appreciate the prestige of hosting them. These putative positives must be interpreted carefully, since host institutions have also reported errors in what visiting students have said [11]. Furthermore, justifying an elective to a low‐resource setting on the basis of disseminating knowledge to that setting may strike some as an impermissibly colonial attitude.

While theoretically possible, it is very difficult to answer the quantitative question of whether these benefits outweigh or justify the burdens imposed. In lieu of a precise quantitative answer, then, a moral‐theoretical evaluation of the structure of harms and benefits in electives will give us a clearer picture of the ethical picture of electives in lower‐resource settings. I aim to show that the application of widely‐used medical ethical principles of distributive justice gives us reasons to doubt the permissibility of electives. As a result, the challenge I lay down in this paper is this: it is the responsibility of those conducting electives (i.e. students) and the institutions supporting them (e.g. medical schools) to demonstrate that a given elective proposal or project is permissible, with specific emphasis on the distribution of risks and potential benefits. To the extent that this is uncertain, those undertaking such electives should strongly consider offsetting the harms their elective may cause.

### 2.3 Classical Utilitarianism

I begin with classical utilitarianism, whose guiding principle is that actions are right when they create the maximum possibly net wellbeing. In the language of Beauchamp and Childress's four principles of biomedical ethics, we might regard classical utilitarianism as requiring we create the best possible distribution of beneficence over non‐maleficence [19]. Strictly speaking, since a maximising consequentialist theory like classical utilitarianism demands the *best possible outcome*, it may be that its requirement is for a medical student to do something entirely different with their elective, or even with their life more generally. It is beyond the scope of this paper to determine the exact demands of consequentialism, but such work has been considered elsewhere [20], and for my purposes here, I will take a narrow view and assume that conducting an elective on a medical degree is consistent with classical utilitarianism.

For classical utilitarians, it would be sufficient justification if benefits of a greater size are realised elsewhere, irrespective of for whom. If the benefits are equal, classical utilitarianism is ambivalent between these states of affairs: it does not matter who gets the benefits if the total benefit is the same in either scenario. This will already strike some as permitting for unacceptable scenarios: extractive electives in which there is overall benefit, but where those in lower‐resource communities are left worse off and those in higher‐resource communities made better off by a greater amount. I think that such a scenario, while theoretically possible, is very unlikely. While we could contrive a thought experiment in which the wealthy enjoy greater benefit from what they take from those with lower resources, in practice it is hard to imagine an elective where this would be so. I therefore think that in practice, in situations where worse off people are further disadvantaged for the benefit of the better off, the principle of diminishing returns indicates that those benefits will not translate into welfare as readily as the costs will translate into harms for the worse off. In short, extractive arrangements where the wealthy take resources from impoverished people are unlikely to confer net benefit. This means that even under a classical utilitarian framework that may be theoretically unappealing to many by virtue of permitting some cases of extractive electives, electives will still be too extractive.

### 2.4 Prioritarianism

Prioritarian views would look even less favourably on scenarios that extract benefit from the worse off for similar‐sized benefits to the better off. For prioritarianism (once called “weighted utilitarianism”) [21], benefits or costs to the worse off should be weighted more heavily, so that the amount of benefit accruing to the better off party would need to be (to some extent) greater than the cost or loss to the worse off party. This puts electives on an even worse footing than they would be under the classical utilitarian lens. Prioritiarianism would not be permissive of extractive electives unless the boon to higher‐resource settings was large enough to outweigh the cost to host communities, according to the heavier weighting of the welfare of the host community.

### 2.5 Egalitarianism

For strict egalitarians, *any* exchange that widens the gap in welfare between the two parties is unacceptable. This would even include Pareto improvements where no one is made worse off and at least one party is made better off [22], and would even go so far as to reject improvements in which all parties are better off, if the better off party's improvement is larger than the worse off party's improvement. A rising tide lifting all boats is permissible only when the lowest “boat” is lifted by at least the same height as those higher than it. Under this framework, then, an elective must benefit the host community at least as much as it benefits the sending community. There are echoes of this view in the electives literature, where there are calls that electives should ensure that they provide *reciprocal* benefits [10, 23].

I do not intend here to advocate for the adoption of any of the above particular models of distributive justice when it comes to electives. In reality, philosophers and non‐philosophers alike will probably mostly operate under hybrid versions of these strictly‐defined principles, with leanings in most or all of the above directions, and in varying degrees. I wish only to note that the simplest way to adapt an elective to satisfy *any* of these moral frameworks is to increase the benefits accruing to host communities. The above application of different frameworks of resource allocation therefore suggests that we should construct electives so that they are beneficial *for host communities*. It is not good enough to point to enriching educational experiences for wealthy medical students; we must couch the moral benefits of electives as accruing mainly to host communities. The literature has only just begun to explore electives from the perspective of host communities, and it is they and what they stand to gain that should be the focus going forward.

### 2.6 A Counter‐Argument From Autonomy

I have already briefly considered the difficulties of establishing an empirical understanding of the distribution of risks and potential benefits in electives. It may yet be shown that the distribution of burdens and benefits is not as I suspect; that it is indeed reciprocal; and that it satisfies some or all of the theories of distributive justice considered above. If this turns out to be the case, it will be a wonderful boon and relief for medical electives, and we can carry on our business as usual in this area. However, I think this has yet to be shown, and that more empirical work to establish these distributions is key.

Outside of the empirical question, there is a strong normative response to the concerns I have laid out so far that electives fail to satisfy principles of distributive justice. The normative argument is that since host communities agree to host elective students from higher‐income countries, it is a matter of their autonomous choice to do so. In its strongest terms, this argument may render my above analysis of harms and benefits paternalistic, and therefore, itself unjust. Proponents of this view will argue that the point is not to assess harms and benefits and then endorse or condemn elective projects according to that assessment; it is to allow institutions to freely choose with whom they conduct their educational business. Doing this is a matter of justice and respect for choice and decision‐making.

To push the argument a touch further, their consent might even be taken as implying an answer to the empirical question above as to where the harms and benefits lie: since people agree to hosting students in their communities, they either believe that the benefits for them outweigh the harms, or they are agreeing as an act of charity or beneficence to the students they host. At any rate, for some, the consent of host communities to engage is sufficient by itself to ground a justification. Absent any threats to consent, like improper information or coercive factors, it is up to the local people, communities and institutions how their time is spent, and it is not up to us to interfere if somebody wants to make a transaction that they think will benefit them, or wants to donate their resources to help others, even if those others are already better off than them overall.

It is worth remembering here that host communities are not monolithic, but comprise different individuals with varied interests. It is therefore possible that what is in the interests of those who decide to host medical students from higher‐income settings may not always reflect the interests of those in the community. It is possible that considerations of prestige or of a desire to be hospitable are what drive people to host such students even if this disadvantages their patients. I nevertheless do not want to take aim at host communities here, and I think that a valuable heuristic is to trust that clinicians will in general have the interests of their communities and patients at heart.

Those for whom autonomy and choice considerations loom the largest may be satisfied that if a host community is happy to proceed with an elective project, that is enough to confer legitimacy across the project and to dispel worries about the distribution of the harms and benefits of the project. My view differs from this: *both* parties need to take responsibility for the distribution of harms and benefits, and it is not sufficient to defer consideration of this to host communities under the morally protective umbrella of autonomy. This is analogous to the way we think about autonomy in medical ethics more generally: a patient's consent and autonomous decision‐making are important, but they do not obviate a doctor's responsibility to consider the risks and potential benefits associated with a given procedure or treatment; autonomy is a necessary but not a sufficient condition for rightful action. The relationship between doctor and patient does not map to the relationship between higher‐income setting and lower‐income setting in an entirely comfortable way, but there is nevertheless a power imbalance in both cases, and there is an ethical imperative for the side with the more power to take at least some responsibility for the consequences of the endeavour.

The autonomy objection, in spite of its intuitiveness in appealing to an anti‐colonial and anti‐paternalistic position, therefore fails to protect medical electives from the accusation that they may cause impermissible harm to host communities. What the autonomy objection does gives us, however, is a steer that there is a greater need to explore how host communities understand the harms and benefits of electives, and how and whether they justify the fact that hosting other students affects their efficiency when it comes to their day‐to‐day tasks.

So far, I have shown that we have reason to be sceptical about the distribution of harms and benefits when students visit lower‐resource settings for their healthcare electives, and that the autonomous choice of host communities to participate in these electives is not by itself sufficient to justify them in light of these concerns about harms and benefits. While more research to determine more precisely how risks and potential benefits are perceived, realised, and conceptualised would be greatly useful in this space, what follows is a proposal for how we can ameliorate the harms/benefits problem in electives with what we know already.

## 3 Practicalities

### 3.1 Offsetting the Harms of Electives

As I have said, a good starting point for dealing with the problems of distributive justice presented by electives is to improve the benefits for host communities. But as I have also said, sometimes the costs for host communities are inherent in the elective project. International electives are one of the most anticipated and fondly remembered aspects of medical training for many people in the health professions, and as such, exhortations to remain in one's home country or community may be ill‐received or ignored by many. This is likely to be the case even though during the worst years of the COVID‐19 pandemic, international electives were cancelled and students had to find creative ways to gain a different learning experience within the constraints of the pandemic. Further, COVID may have introduced a range of difficulties for students on international electives that persist today [24]. Despite this, appetites for international electives appear to be strong. So, while refraining from going to a lower‐resource setting might be the best option, I will explore a way that health professions students can make electives of this sort more ethically permissible. Short of throwing out the elective project altogether, then, medical students could do, or commit to doing, something for that community or some other community going forward, as a way to bolster the justification of the overall elective plan. They should offset the harms of their elective.

Offsetting means compensating for harms caused by doing an equal or greater amount of good somewhere else. It is a concept most commonly found in the context of emissions that exacerbate global warming: the argument goes that one can legitimise the performing of actions that result in greenhouse gas emissions by also performing some *other* action that causes some climate benefit of an equal or greater degree than the harm. Most directly, this might be planting trees (or paying an organisation or charity to plant them) that will capture an amount of carbon equal to the emissions caused by the first action.

Offsetting need not be direct, and schemes of this sort may be employed as defences against accusations of free‐riding. I am not free‐riding on society's willingness to build streetlights in my neighbourhood that I benefit from, since I pay taxes into the system that provides streetlights and other such amenities. I am not required to pay directly into the “streetlight system” by going out and erecting streetlights myself – it is enough that I contribute in some other way [25].

Maximising consequentialist theories like utilitarianism may still condemn offsetting schemes, since in many cases these theories would dictate that one should perform the offsetting behaviour whilst simultaneously refraining from the harmful behaviour that led one to consider offsetting in the first place. According to such theories, instead of imposing a harm and then compensating for it later, the compensation should be offered up anyway, and the harm avoided. An offsetting scheme may therefore only be permissible under this framework if it happens that the best course of action involves that imposition of harms in the first place. This may be the case if the learning that a health professions student gets from their elective is required for them to achieve their maximum possible impact. Most other, more moderate theories may, however, leave room for offsetting, as long as the harms being offset are acceptable and do not in themselves violate some important rule or principle to an unacceptable degree.

### 3.2 How Can the Harms of an Elective Placement Be Offset?

There is then the question of *how* a person can actually effectively compensate for the harms their elective has caused. In the previous section, I briefly discussed a distinction between paying someone to plant trees, and planting trees oneself, as a means of offsetting emissions. This distinction may capture the primary two ways in which a health professions student might offset the harm caused by their elective project. First, they might do this in the form of a monetary payment. Where exactly this payment should go may again rely on moral theory: arguments from a duty to compensate and/or justice may require that the funds go to the specific community that has been harmed, or at least a similar one. On the other hand, broader consequentialist theories might suggest just putting the funds wherever they will do the most good, irrespective of the relevance of this donation to the specific harms caused by the elective. I will not attempt to answer this question more precisely here: and indeed, for students of healthcare who will also be studying healthcare ethics, the puzzle of resolving this conflict between reciprocity and beneficence may be a useful moral‐theoretical exercise.

The same puzzle applies to the second mechanism of offsetting, which would be for a student to perform charitable work with the skills they have gained as a medical practitioner. The offsetting clinician may consider whether they should aim to maximise the good they can do with their skills by taking a broad view of where they might use them, or whether the compensation duty obliges them to target their efforts towards communities that are similar in some way to the one in which they undertook their elective.

There is also a temporal distinction – students may decide to do the offset before/during the placement, or later in life. They may be able to have more impact later in life across both dimensions, but run the risk of forgetting the importance of the offset if it is delayed until later. The existence of this concept of “ethical drift” is both intuitive and supported in the literature [26]. Notwithstanding these more detailed questions, it seems reasonable to consider the two principal means of offsetting to be financial or direct work.

### 3.3 Who Will Organise the Offsetting Programme?

How this offsetting is to be administered is the next question, and one that may depend on the reader's position and role. Medical educators might consider encouraging their students to consider this problem, and advocate that they offset accordingly. Students themselves reading this may get the ball rolling on their own without instruction from their local educators. Host institutions may also consider encouraging their guests to consider these problems more carefully. Those from any of the above groups may wish to develop the idea further by implementing a scheme to organise offsetting more systematically. We could even generate a scoring/credit system that aims to classify the burdens imposed and give students an idea of what they should do to counterbalance these, either before the elective or at some later point in their careers. Not only would this encourage acts of beneficence and/or reciprocity, but it would bring into sharper relief the fact that these burdens are imposed. This means that, even if one thinks the idea of moral credit is distasteful and overly consequentialist, it nevertheless has useful benefits in terms of advocacy and raising awareness about this problem.

This proposal is workable at a large scale. For a start, it only takes individual health professions students reading this paper and deciding to take action for it to become an effective programme. Moving beyond that, educators involved with pre‐departure training can include this idea in their teaching sessions and materials to encourage further uptake. A useful future piece of work would be for academics and sending institutions to work with host institutions to establish what the actual tariffs might be, and develop a framework for students to calculate roughly appropriate offsetting amounts for their individual elective endeavours. These frameworks can then be modified and shared between institutions. Beyond the institutional level, governing bodies like the United Kingdom's General Medical Council may also have a role to play, both in publicising and advocating for elective offsetting, and in participating in the refinement of the frameworks according to which offsetting amounts can be determined. A central database or record of offsetting efforts could be kept, to help future health professions students to consider their own obligations in this space.

### 3.4 Who Should Do the Offsetting?

There is then the question of who exactly should bear this additional cost of a student's elective. The most obvious candidate is the student themselves. This is consistent with the individualised nature of the elective concept: as the name implies, electives are a matter of personal choice, so the costs of the learning experience should be borne by the student. This is an extension of the current system, wherein the student generally pays for all the costs associated with the experience.

Electives are expensive, however [27], and introducing an additional cost will only exacerbate the existing problem that only the wealthiest students can access certain elective opportunities. Arguing that the cost is optional will not do; to the extent that we want students to take the possible ethical obligation to offset seriously, it will exacerbate this inequality.

My response is that this is the lesser of two evils. Exacerbating inequality at home is unfortunate, but if that is the cost of compensating for some wider inequality between nations, this still seems like a net improvement. Any putative improvement that has a cost attached is likely to exacerbate inequality somewhere down the line, since differential abilities to meet those costs seem to be a fundamental nature of the human condition. We can sympathise with those students whose ability to afford an elective may be diminished by the proposals this paper makes to improve electives, but it is not clear that that is a reason not to improve electives in this way. Since justice is the apposite consideration in both cases, this is not a tension between principles – it is simply the same principle pulling in two different directions, and it seems intuitive that the bigger injustice is the extractive elective whose victim is those in lower‐resource settings.

Other options would be for institutions to cover these costs, i.e. the medical schools sending the students. This would reduce the inequality problem at home to an extent. It is beyond the scope of this paper to discuss whether medical schools would be able or willing to do this, but I will make some philosophical comments on this.

Medical schools paying to offset the harms of electives would have the advantage of there being a guaranteed payment. Instead of leaving this issue to the consciences of the individual students, we would have greater assurance that efforts were being made to repair the harms of electives. It would be important to ensure that students understand what the payment is intended to do, and that they are still expected to conform to high standards of ethical behaviours while they are away: it is not a blank cheque that permits them to do anything they like. I discuss this problem in more detail in the next section.

A disadvantage might be that there is some moral good in having students do the work of understanding and organising the offsetting concept for themselves. If offsets are paid at the institution level, we run the risk of failing to educate students about the moral harms of electives and the reason for the offsetting scheme's existing in the first place. At the same time, it could foster some interesting discussion and criticism of the offsetting scheme itself.

### 3.5 What Happens to the Money?

One objection is that the host institution will misuse the money, or otherwise fail to effectively use it to offset the harm caused by the elective. For example, if one of the harms is an increased risk of staff burnout when those staff must use additional time to supervise visiting elective students, we might be suspicious that those staff will ever see the benefit of the additional payment that goes to the institution.

This is a legitimate concern, insofar as it is surely correct that the people – be they staff, patients, or someone else – who incur costs from hosting students will not always be the people who benefit from the offsetting payment, either in cases where the payment is monetary, and especially where the offsetting is done through work later on.

The response to this was touched on in the discussion above about how exactly an offsetting scheme should work from an ethical perspective: it may be preferable that the goods of offsetting find their way directly to those people who were harmed, but alternatively, we might think that benefitting the same *community*, if not the same exact *individuals*, is sufficient for an offsetting scheme to be worthwhile. The latter seems plausible, since it is surely better to compensate *somebody* for the harm caused than for the affluent health professions student to simply keep that money for themselves. Finally, I think that cases where we do not think that at least some attempts will be made to use at least *some* of the money for social good are likely to be rare.

## 4 Delineating Which Harms Can Be Offset

A serious objection to offsetting schemes for electives is if health professions students interpret the offsetting scheme as giving them licence to impose further burdens or to be less vigilant about other ethical concerns. As a simplified example, imagine a student who spends time seriously considering the burdens their elective will impose, and constructs an offset plan comprising some financial donations, and a commitment to conduct further healthcare work in a lower‐resource setting once they qualify. Imagine this student is then faced with a classic elective dilemma when they are away, but they dismiss it on the basis that they will offset the burdens of their elective anyway, and decide to proceed with attempting to provide care that they know falls outside their clinical competence. This is an unpalatable situation.

Evidence from the literature may support the existence of this problem. The phenomenon of “moral licensing” occurs when individuals – and groups [28] – regard themselves as having recently performed morally good actions, their inclination and willingness to perform morally bad actions increases [29]. They also feel more licensed to do bad things if they have simply *recalled* doing good things. The moral licensing effect even appears when people are asked to describe themselves positively by writing down their positive character traits. The opposite effect has also been found, so that people who describe themselves badly are consequently more likely to do good things [30]. If moral licensing means that someone will use their offsetting credits to justify behaviour we regard as unacceptable, then the offsetting scheme may be a cure that is worse than the disease.

Importantly, the fact that we *do* act in this way as a matter of psychological fact (if such a strong word can be used to describe these findings) is little or no indication of what we *should* do as a matter of normative principle. The hardest‐nosed maximising consequentialist theory, for example, would require that we do what we can to see past these quirks of our psychology and ensure that we perform the best action anyway. Similarly, deontological theories may ground a duty to compensate others for harms done to them [31], but it does not follow from this that wilfully causing harm is permissible when the action is accompanied by an intention to compensate.

Similarly, the possibility of offsetting harms should also not stand in the way of wider efforts to make elective placements more reciprocal, for example the “Ethical Educational Placements” idea advocated by Ahmed et al. [32] But an important distinction is that reciprocity is usually about the mutual exchange of goods, so to couch elective ethics *solely* in terms of reciprocity is, I think, to fail to recognise the harms that are caused to host communities by electives. Reciprocity is an ideal standard, but in the meantime, harms must be compensated for, and that is where offsetting comes in.

Educators may be able to resolve this by ensuring that pre‐departure training – a crucial aspect of ensuring an ethical elective experience [33, 34, 35, 36] – describes appropriately the relatively narrow realms in which the offsetting system should work. This may involve circumscribing the precise types of harms that are candidates for offsetting. As a rough preliminary sketch of this, we might say that direct harms to patients or violations of their autonomy are not candidates for offsetting, but disparate harms to the host community generated by consuming their resources (in terms of staff time and so on) *are* candidates for offsetting. We might also wish to add the non‐consequentialist idea that, all other things being equal, it is better to avoid imposing the cost in the first place than to impose it and offset it. Considerations like these may be theoretically robust to varying degrees; but public acceptability matters, and so steps to make offsetting schemes more appealing (or less unappealing) to practitioners, educators, the general public, and host communities, are *pro tanto* positive steps.

Ideally, the concept of offsetting these burdens will cause health professions students to *widen* their sphere of moral consideration when pondering the ethical aspects of electives. Indeed, the psychological situation may give us some practical advantage in encouraging people to take up an offsetting scheme: the more we talk about the harms of electives, the more likely it may be that a person doing such an elective feels compelled to compensate for this harm. In this way, the phenomenon of moral licensing may be used to positive effect – if students are made fully aware of the harms they may be causing, this may cause them to perform compensatory actions that they otherwise would not have performed.

I will leave open the question of whether educators should insist that their students engage with a compensatory offset system like the one I have described. Notwithstanding the difficulties associated with policing such a system (especially if a student commits to offsetting their elective's harms later on in life), it may still be possible to require to declare that they will offset. This may generate constructive compensatory action on the part of the student, but it may have the effect of becoming a perfunctory tick‐box exercise. The ideal situation would be for students to be educated in the moral problems at play, introduced to the concept of offsetting, and make a moral decision for themselves. Leaving the moral decision‐making with the student like this would have the ancillary benefit of providing a further opportunity for students to sharpen their moral reasoning skills. At the same time, students are relative newcomers to the practice of medical‐ethical reasoning, and so given the seriousness of global health disparities and the possibility of their electives exacerbating these, it may be appropriate to take the decision out of their hands. Importantly, my proposal will not solve all the problems of medical electives, and those undertaking them should still undergo the usual suite of training to assist them in navigating the ethical issues that can arise, reminding them of the need for respect, candour, probity, and other virtues required of medical professionals.

## 5 Conclusion

Electives have been around for a long time, and are a popular and exciting part of the curriculum for health professions students. There have been laudable efforts in more recent years to implore educators and students alike to consider electives that are of mutual benefit, and I think that, where this can be achieved, this is a positive step. However, the moral‐theoretical analysis of electives according to different theories of distributive justice gives us reasons to fear that electives where mutual benefit is *not* achieved may be the norm, and may continue to be so. Therefore, the offsetting scheme I am advocating here should be considered a proposal of harm reduction: electives are here to stay, and this is one way to improve their ethicality.

## Ethics Statement

The author has nothing to report.

## Conflicts of Interest

The author declares no conflicts of interest.
